# Smart textiles for multimodal wearable sensing using highly stretchable multiplexed optical fiber system

**DOI:** 10.1038/s41598-020-70880-8

**Published:** 2020-08-17

**Authors:** Arnaldo Leal-Junior, Leticia Avellar, Anselmo Frizera, Carlos Marques

**Affiliations:** 1 Graduate Program in Electrical Engineering, Federal University of Espírito Santo (UFES), Fernando Ferrari Avenue, Vitória, 29075-910 Brazil; 2 I3N and Physics Department, Universidade de Aveiro, 3810-193 Aveiro, Portugal

**Keywords:** Quality of life, Optical sensors, Optoelectronic devices and components, Polymers, Biomedical engineering

## Abstract

This paper presents the development and application of a multiparameter, quasi-distributed smart textile based on embedded highly stretchable polymer optical fiber (POF) sensors. The POF is fabricated using the light polymerization spinning process, resulting a highly stretchable optical fiber, so-called LPS-POF, with Young’s modulus and elastic limits of 15 MPa and 17%, respectively. The differential scanning calorimetry shows a thermal stability of the LPS-POF in temperature range of 13–40 °C. The developed sensors are based on the optical power variation, which results in a fully portable and low-cost technique. In order to obtain a multiplexed sensor system, a technique based on flexible light emitting diodes (LEDs) on–off keying modulation is applied, where each LED represents the response of one sensor. The smart textile comprises of LPS-POF and three flexible LEDs embedded in neoprene textile fabric. The performance of the system is evaluated for temperature, transverse force and angular displacement detection at different planes. The sensors presented high linearity (mean determination coefficient of 0.99) and high repeatability (inter-measurement deviations below 5%). The sensor is also applied in activity detection, where the principal component analysis (PCA) was applied in the sensors responses and, in conjunction with clustering techniques such as k-means, indicate the possibility of detecting basic activities such as walking, sitting on a chair and squatting.

## Introduction

The internet of things (IoT) concept mainly relies on the wireless connectivity of devices, which place demands towards a constant evolution in wireless systems and their miniaturization. Such evolution resulted in many developments in industry^1^, smart cities^2^ and remote healthcare^3^ applications. The latter plays an important role nowadays due to the demographic growth in conjunction with the population ageing^4^, where there is an increasing demand on smart systems for remote healthcare^5^. In this scenario, it is desirable the continuous monitoring of human activities for remote assistance, which include diagnosis, transportation in case of emergencies and monitoring of patient’s health condition^6^. The remote health monitoring leads to advantageous features such as reduction in the treatment cost and hospital (and clinical facilities) occupation^5^. As another feature of the home monitoring, “staying-at-home” factor brings important emotional and psychological advantages for the patient due to the possibility of performing their daily activities and the sense of independent growth in the community^7^.

As a popular technology for the remote health monitoring, different wearable sensors have been proposed for the assessment of multiple parameters of the user^8^. The parameters for wearable sensors in human monitoring include the movement assessment and analysis, body temperature, interaction forces/pressures (between human and objects), humidity and physiological parameters, including heartbeat and breath rates^9^. It is also worth to mention the assessment of additional parameters in some cases such as arterial pulse, electromyography signals and pulse oximetry^8^.

As a growing approach for wearable sensors systems, smart textiles offer the advantages of higher transparency between the sensor and the user, i.e., the sensor system is lightweight, compact and does not inhibit the user’s movements^10^. The application of compact and embedded sensors in smart textiles and their advantages of easy installation and removal have a positive effect in the system’s usability^5^. In light of these advantages, the developments on the flexible electronics have enable the development of flexible wearable sensors such as the ones summarized in previously reported reviews^9^. The smart textile technologies continue to point towards an even higher miniaturization, low energy consumption and wireless connection, which are well-aligned with the requirements of the IoT devices^11^. Such advances include resistive sensors embedded in fabrics patches^12^ and dual core microfibers for capacitive measurements^13^, including different embedment methods and circuitry^14^.

Optical fiber sensors have experienced a large growth in many fields of applications, including industrial^15^, structural health monitoring^16^ and healthcare^17^. In such applications, the optical fiber sensors offer advantages such as compactness, lightweight, potential for multiplexing capabilities, intrinsically safe operation and electromagnetic interference immunity^18^. Such advantages are especially important for wearable applications, where it is possible to obtain compact sensors with safe operation (since there is no electrical currents in the sensor’s head) and immune to electromagnetic interferences. Such interferences occur due to assistive devices (especially for users with health impairments) of the user as well as devices that emits electromagnetic waves, commonly used nowadays with the widespread of portable technologies.

These advantages motivate the development of photonic-integrated textiles, which, in their first reports, begun as clothing accessory or signaling devices^19^. However, with the widespread of optical fiber sensors, the so-called photonics textiles are applied on the body temperature sensing^20^, breath and heart rates^21^. Many of the reported sensors are based on fiber Bragg gratings or other wavelength-encoded sensing approaches, which have a high precision and immunity to light source power deviations, but need an optical spectrum analyzer or an optical interrogator, where such devices are generally bulk and non-portable with high cost (when compared with other sensing techniques)^17^. In addition, such sensors employ silica optical fibers, which, despite their lower optical loss, have a brittle nature with low impact resistance and strain limits^18^. It is also noteworthy that in case of breakage, the glass fiber may puncture the user^22^. In order to overcome these drawbacks, advances in the polymer processing, preparation and fabrication have enable the development of polymer optical fibers (POFs), which present higher strain limits, flexibility and impact toughness. Their rugged surface also make POFs easier to incorporate in textiles, where such features have been demonstrated in many works for wearable sensors for human health assessment^17^. Furthermore, the smart textiles with POF sensors are mainly based on the intensity variation sensing principle, in which portable sensors with lower cost (when compared to wavelength-based sensors) are achieved.

As a drawback of the previously proposed systems, the intensity variation principle is sensitive to light source power deviations, leading to the necessity of techniques for compensation of light source power deviations^23^. Moreover, these sensors are not multiplexed, as they are mainly based on point detection of a single parameter, if more than one point or parameter needs to be simultaneously detected, there is the need of increasing the photodetectors, which leads to proportional reduction of the system’s portability and increase of wearable system cost. Although POFs have Young’s modulus one order of magnitude lower than silica fibers (leading to higher flexibility), the Young’s moduli of commercially available POFs are in the range of 1–4 GPa^18^, which are some order of magnitude higher than the conventionally applied materials in fabrics/textiles (in the range of tens of MPa)^24^. Thus, the integration of commercial POFs (with diameters of 0.5–1.0 mm) in a textile may lead to a lower flexibility and freedom of movement of the clothing.

This paper presents a novel POF-integrated optoelectronic smart textile, which can overcome the limitations discussed above. The POF used in this work is fabricated through the light polymerization spinning (LPS) process, in which a mixture of monomers are polymerized with UV light, resulting in a higher degree of customization for the so-called LPS-POF^25^. The proposed LPS-POF has Young’s modulus more than 100 times lower than commercial POFs, which is even lower than the elastic modulus of cotton and other textile/fabric materials. In addition, a multiplexing technique for intensity variation sensors based on side-coupling between the light source and the LPS-POF was applied to overcome the other limitation of the current proposed smart textile, i.e., the multipoint and multiparameter sensing^26^. The proposed LPS-POF integrated multiparameter smart textile also takes advantage on the developments on flexible electronics, where light emitting diodes (LEDs) in flexible substrates are used as light source in order to further enhance the system’s flexibility, usability and transparence. The multiplexed system enables the measurements on multiple parameters in different parts of the user’s body, whereas the high flexibility of the materials enable not only higher flexibility of the sensors, but also does not inhibit the user’s natural movement. Thus, the proposed system was tested in the assessment of movement at different planes, temperature, interaction force between the user and the environment and activities monitoring.

## Results

### Multi parameter LPS-POF smart textile development

The POF is fabricated by the LPS process in which there is a combination of monomers and additives instead of the extrusion of a polymer preform, conventionally used in the optical fiber fabrication. This process has intrinsic advantages when compared with the preform extrusion method such as higher customization and repeatability.

The LPS-POF has a diameter of 580 µm ± 30 µm with a core refractive index of 1.54, whereas the fiber cladding (with 20 µm thickness) has a refractive index of 1.45. It is worth noting that such a high diameter and refractive index difference leads to a large multimode behavior of the fiber. Although the large number of modes harms the application of many spectral-based sensors such as fiber Bragg gratings (FBGs), it does not inhibit the use as intensity variation-based sensors. In addition, as an acrylate-based fiber, the LPS-POF has lower optical losses at the visible wavelength, especially at 650 nm, where the optical loss is 4 dB/m. Thus, flexible LEDs at the visible wavelength range were used.

**Figure 1:**
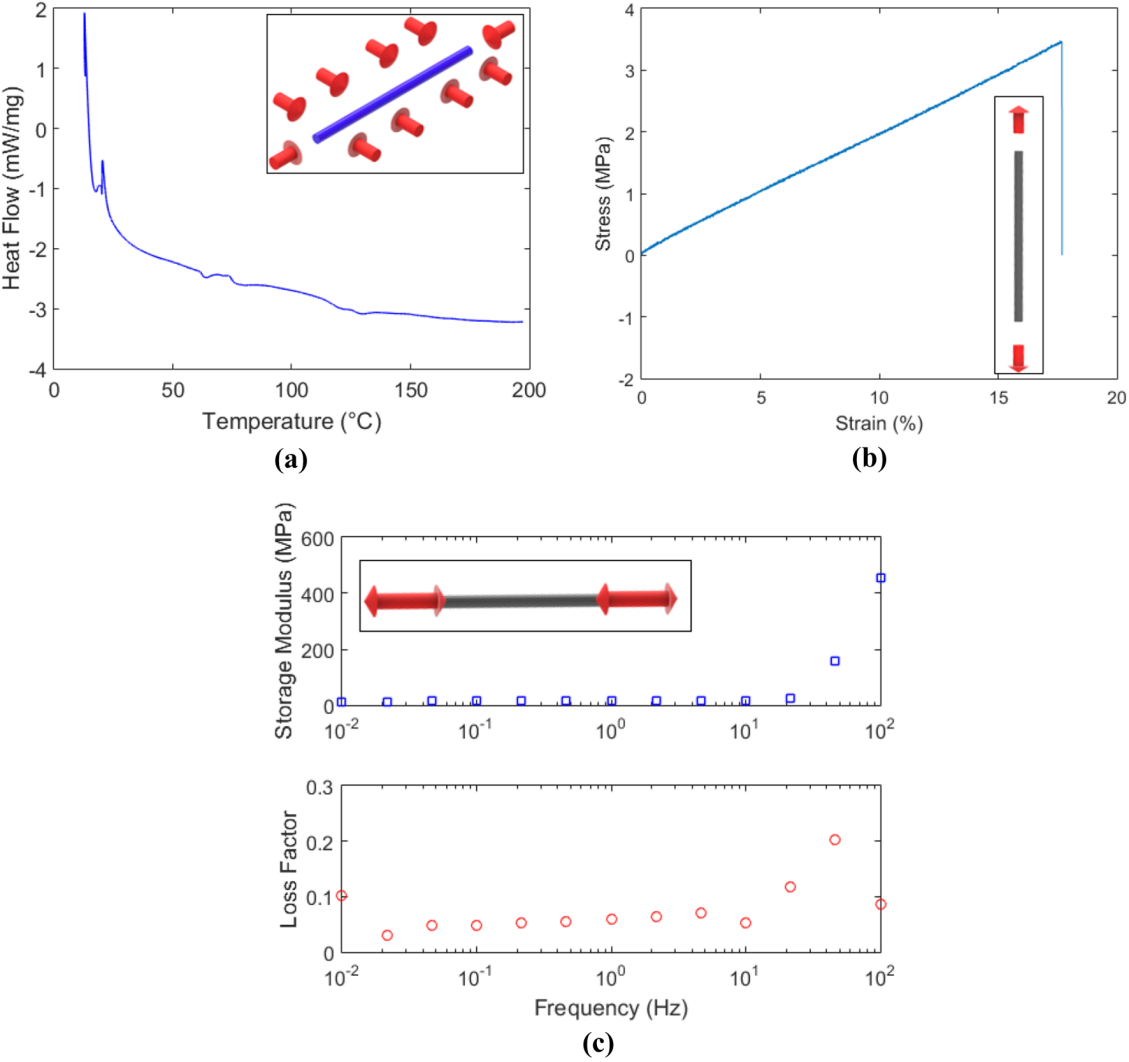
Characterization of the LPS-POF: (**a**) DSC results, (**b**) Stress–strain curves in static tensile tests and (**c**) Storage modulus and loss factor in DMA for different frequencies. Figure insets show a schematic representation of the thermal and mechanical loadings in the fiber.

Regarding thermal and mechanical properties of the proposed fiber, the operation limits and the optical fiber behavior at different conditions were tested through the differential calorimetry scanning (DSC), tensile tests and dynamic mechanical analysis (DMA). The DSC test was performed at a temperature range of 13–200 °C using the DSC Q200 (TA Instruments, USA), where the heat flow variation of the LPS-POF is analyzed on the predefined temperature range as depicted in Fig. 1a. For the mechanical characterization, a universal tensile test machine (Biopdi, Brazil) was employed. In the stress–strain curve shown in Fig. 1b, material parameters such as Young’s modulus and elongation at break are estimated. However, the polymer is a viscoelastic material, which presents time-dependent properties^27^. For this reason, the DMA was employed in the LPS-POF to analyze the dynamic modulus, represented by the storage modulus and loss factor (as discussed in previous works^28^), at oscillatory movements with different frequencies. In DMA tests, an oscillatory load with controlled displacement and frequency is applied on the fiber sample, the variation of the dynamic properties, namely the storage modulus and loss factor, as a function of the frequency in the range of 0.01–100 Hz. This range was chosen based on the proposed application, i.e. human physiological parameters and movement/activities assessment, in which the frequency of movement is below 100 Hz^29^. Figure 1c shows the results obtained in the DMA tests for the frequency analysis. The insets in Fig. 1 show a schematic representation of the thermal and mechanical loadings that the fiber is subjected in each characterization.

Regarding the DSC results, there is a large endothermic baseline change, related to differences between the heat capacity of the sample and the reference in the DSC experiments (see Methods section). It is also worth noting a change in the curve slope at about 40 °C, which is related to the material’s glass transition temperature^30^. Additionally, the melting point of the fiber is represented by an endothermic peak in the heat flow. However, such peak was not observed in the temperature range employed, which indicates that the LPS-POF melting point is higher than 200 °C. Thus, the thermal degradation, which generally occurs after the polymer melting, also does not appear in this temperature range. Nevertheless, there is a small exothermic peak at about 80 °C that can be related to the LPS-POF crystallization. Therefore, the thermal characterization of the LPS-POF indicate its suitability on small temperatures as the ones for on-body application (circa 36 °C) or room temperature assessment. For the mechanical characterization of the LPS-POF, the stress–strain curve in Fig. 1b shows a large linear range for the fiber of about 17% with a Young’s modulus of 15.0 MPa. In addition, the dynamic analysis presented in Fig. 1c indicates the feasibility of the proposed LPS-POF on dynamic conditions in frequencies up to 21 Hz, such as in human movement. In the range of 0.01–21 Hz there is no significant variation in the material’s storage modulus (elastic component) and loss fact (ratio between the elastic and viscous component). However, in higher frequencies, there is a sharp increase of the LPS-POF storage modulus, reaching its maximum value of 450 MPa at 100 Hz. As the sensitivity of optical fiber sensors for mechanical parameters assessment is proportional to the fiber Young’s modulus, such increase in the fiber Young’s modulus leads to lower sensitivity for stress-related parameters sensing at oscillatory frequencies of 100 Hz. In addition, the variation of the sensitivity also results in a nonlinear behavior of the sensor.

**Figure 2:**
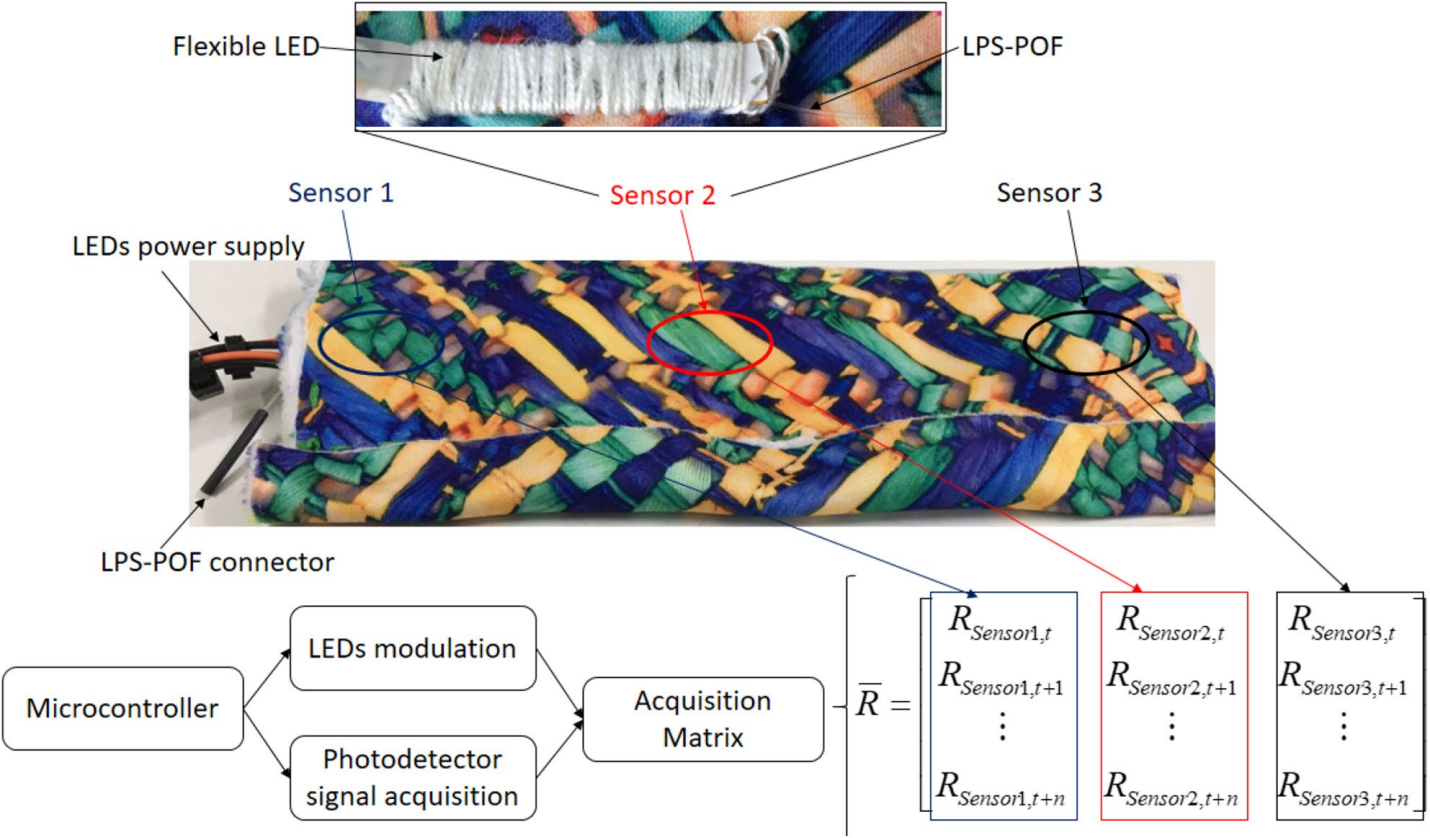
A picture and schematic representation of the proposed multifunctional smart textile. Figure also shows the acquisition matrix with the responses of the sensors.

The LPS-POF has promising mechanical features and properties for mechanical sensing with the possibility of embedding on textiles without changing the textile stiffness, which makes it suitable for multiparameter sensing in smart textiles. In order to achieve such multiparameter sensing using low cost intensity variation-based sensors, a multiplexing technique was employed in which there is a side coupling of the light source in the fiber and the end faces of the fiber are connected to photodetectors. In this case, the light source is a LED in flexible substrate to ensure a high flexibility to the system embedded in the textile fabric. Then, an on–off keying (OOK) modulation is applied to each flexible LED in a way that only one LED is activated at time. Thus, there is no simultaneous activation of each LED, where the position of each LED represents a sensor point in the fiber. For this reason, a quasi-distributed sensor system is achieved using only one fiber and photodetector, where the number of sensors in the system is equal to the number of LEDs side-coupled to the fiber. A microcontroller FRDM-KL2Z (NXP, Netherlands) is used for the activation signals to the LED and for the acquisition the data from the photodetector IF-D92 (Industrial Fiber Optics, USA) through its 16-bit analog-to-digital converter. As another key process in the microcontroller, there is a synchronization between the LED activation and the data acquisition from the photodetector, where the data acquired is positioned in a matrix (as shown in Fig. 2). Each column represents the active LED and the lines are the data acquired through time. In this proof of concept, three flexible LEDs were used (as also shown in Fig. 2) and the matrix has three columns (for LEDs 1, 2 and 3). The data at each column is the photodetector signal when each LED is active, i.e., the data in column 1 is the one gathered when LED 1 is active, in column 2 when LED 2 is activated and so on. The proposed multiplexing technique not only allows the position assessment of mechanical disturbances in the LPS-POF, but also the multiparameter sensing by characterizing the sensors with respect to each of the desired parameter^26^. In addition, the system has scalability and a higher number of sensors can be used with few centimeters distance between them^31,32^.

Thereafter, the sensor system (comprised of the LPS-POF and the flexible LEDs) is sewed between two layers of a neoprene textile fabric. Figure 2 presents a picture of the proposed smart textile, where the optical fiber connector and the cables for the LEDs supply are also shown. Both of these cables connect in the microcontroller and its shield with circuit the board for the photodetectors and LEDs activation. Thus, the proposed system is fully portable solution in which the acquisition module comprised of the two photodetectors and the microcontroller can storage the data in a SD card or send it wirelessly using a Bluetooth connection to a host device (such as computer, tablet or smartphone) with acquisition frequency of 100 Hz. As an important parameter for wearable applications, the system can be considered a lightweight solution due to its total weight of 400 g, including microcontroller, photodetectors, LEDs and batteries. In addition, the proposed smart textile has low power consumption with an average consumption of 150 mA was obtained for the whole system (i.e., photodetectors, microcontroller and LEDs), which enable an autonomy as high as 10 h using commercially available 10,000 mAh power banks.

The proposed system is characterized and validated with respect to different movement and physiological parameters. First, different temperature profiles are applied on the sensors and their temperature responses are evaluated for the possibility of on-body temperature sensing. Then, controlled pressures are applied at each sensor in order to detect the interaction pressure between the user and the environment that can be used for activity monitoring. In addition, different movements of bending and torsion at different planes are applied on the textile in order to verify the system’s capacity for movement analysis applications. Finally, the smart textile is positioned on the lower back of a volunteer and the sensors responses are analyzed for commonly activities on daily routine, including walking, squatting and sitting, where the signals are analyzed using the Principal Component Analysis (PCA) in conjunction with clustering technique (such as k-means), which leads to the activities detection.

### Smart textile responses to thermal and mechanical parameters

**Figure 3:**
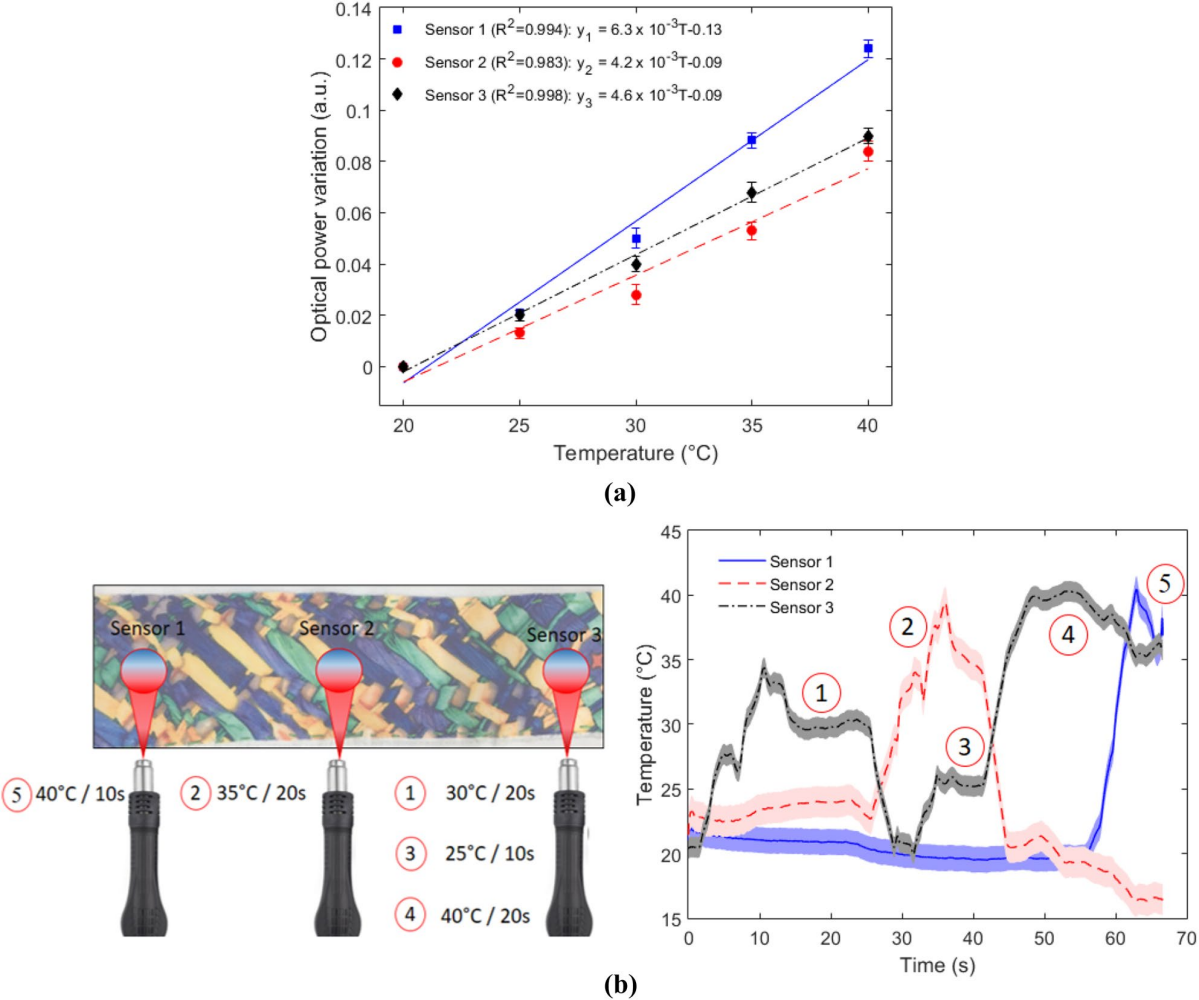
Temperature analysis of the LPS-POF embedded textile. (**a**) Temperature characterizations. (**b**) Temperature responses of each sensor for different heat spots.

The LPS-POF embedded smart textile was tested in different conditions in order to verify its suitability on measuring multiple parameters, where the multiplexing technique based on the LEDs modulation enables the simultaneous measurement of multiple parameters in multiple points on the LPS-POF. In this case, the tests were performed in the textile with three measurement points. Figure 3 presents the temperature response of each sensor, where the tests were performed in a range of 20–40 °C due to both the intended application (room temperature monitoring and on-body applications) and the thermal restrictions in the fiber presented in Fig. 1a. The temperature characterization of each sensor (Fig. 3a) shows a linear behavior of all sensors in the mean and standard deviations of five tests, where the sensors 1 presented the highest temperature sensitivity. The temperature increase in the LPS-POF leads to refractive index variation due to the thermo-optic effect, which results in variations in the transmitted optical power, the differences in the sensitivities can be related to anisotropy in the fiber material^33^. From the linear regressions obtained in the sensors characterizations in Fig. 3a, it is possible to estimate a temperature distribution in the textile, as shown in Fig. 3b. In this case, a thermal blower is positioned in different locations along the fiber, leading to both distributed and concentrated temperature increase in different regions. The capability of sensing the temperature distribution in the textile was demonstrated, where the sensors also presented low cross-sensitivity between them, demonstrating the feasibility of the proposed multiplexing technique. Regarding the temperature responses, the solid lines are the responses of the sensors applying the linear regressions, whereas the shaded lines are the temperature uncertainty of the sensors, considering the standard deviations on the characterizations shown in Fig. 3a. The positions and temperatures of the heating spots on the optical fiber are also presented in Fig. 3b, where it can be seen a temperature increase starting in the Sensor 3 and ending in Sensor 1. Thus, the proposed sensor system can be used for body temperature monitoring and can be applied in the interface between the user and an assistive device for microclimate assessment.

**Figure 4:**
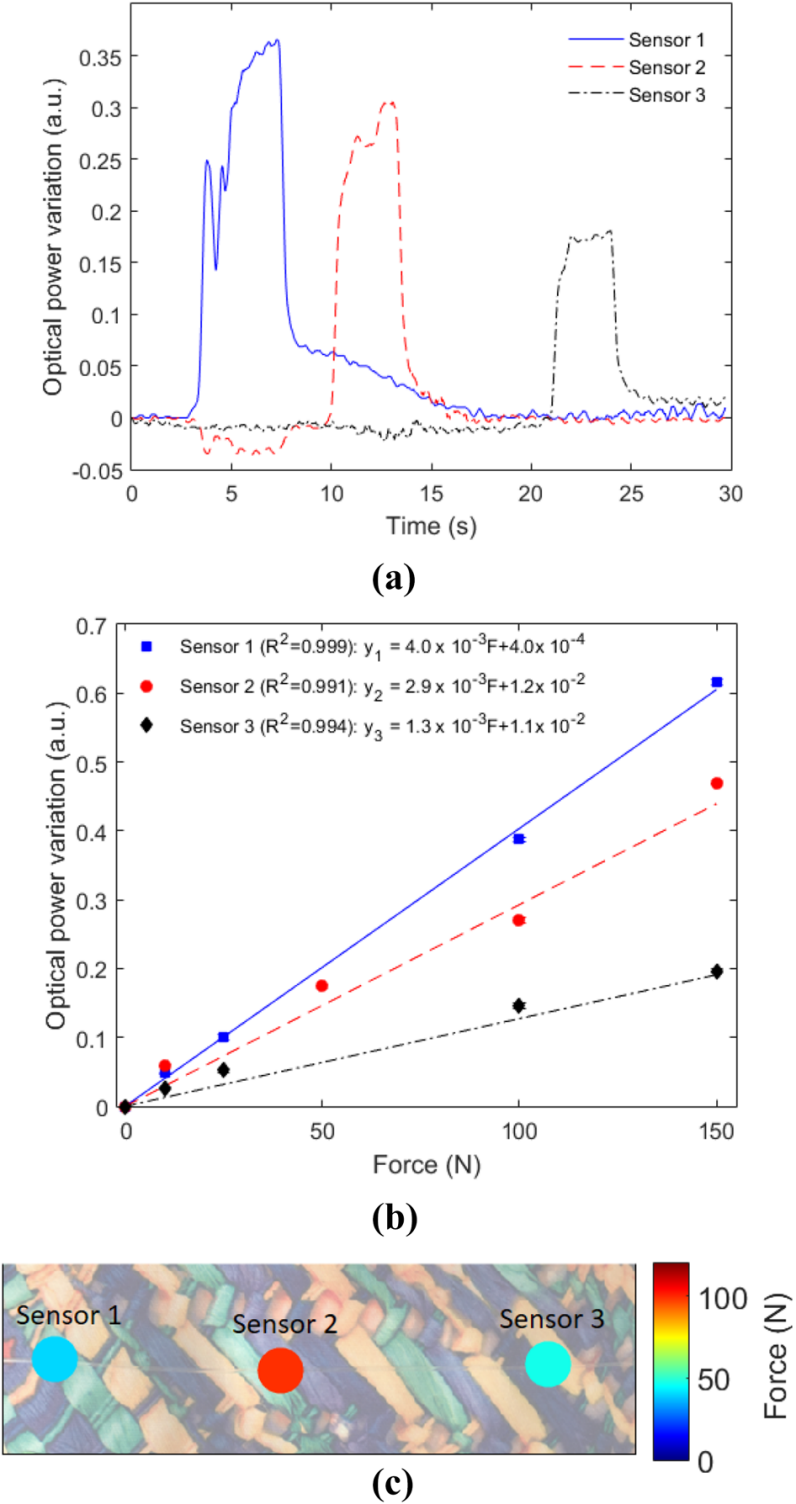
Force analysis of the LPS-POF embedded textile. (**a**) Transmitted optical power attenuation as function of time for forces applied at different sensors. (**b**) Force characterizations. (**c**) Force map from the sensors responses with a force applied on the textile.

Thereafter, different forces are applied at each sensor to obtain their responses at transverse force conditions, which can be correlated to the applied pressure by considering the area of each sensor. In order to show the low crosstalk between sensors, Fig. 4a shows the sensors responses as a function of time for a force of 100 N applied at one sensor at a time (starting from Sensor 1). It is possible to observe the low cross-sensitivity between sensors, i.e., when the force is applied directly on one sensor, there is no significant signal variation in the others, showing the feasibility of the proposed LED modulation multiplexing technique for intensity-based sensors. In Fig. 4b, the force characterization is presented, where high linearity of all sensors and low standard deviation between tests is shown. By applying the linear regression in the sensors responses, it is possible to obtain a pressure/force map of the interaction between the sensor and the user (or environment), as shown in Fig. 4c. In this case, a rectangular object (resembling a chair support) is positioned on the textile and the force map is presented in Fig. 4c, which indicates the possibility of using the proposed smart textile with a mesh of sensors to evaluate the interaction pressure between the user and the environment, such as chairs and beds. Such evaluation is important to provide a remote monitoring of the user’s activities and prevent pressure ulcers.

Comparing the force and temperature responses, the sensors presented higher optical signal variation in the force tests when compared with the temperature tests, see Figs. 3a and 4b. The signal variations are more than 3 times higher in the force tests than in the temperature experiments, which results in a higher signal-to-noise ratio of the force response (compared with the temperature response). Thus, if the sensors responses as function of time are compared, as shown in Figs. 3b and 4a for temperature and force responses, respectively, there is a higher stability of the optical power variation in the force response.

**Figure 5:**
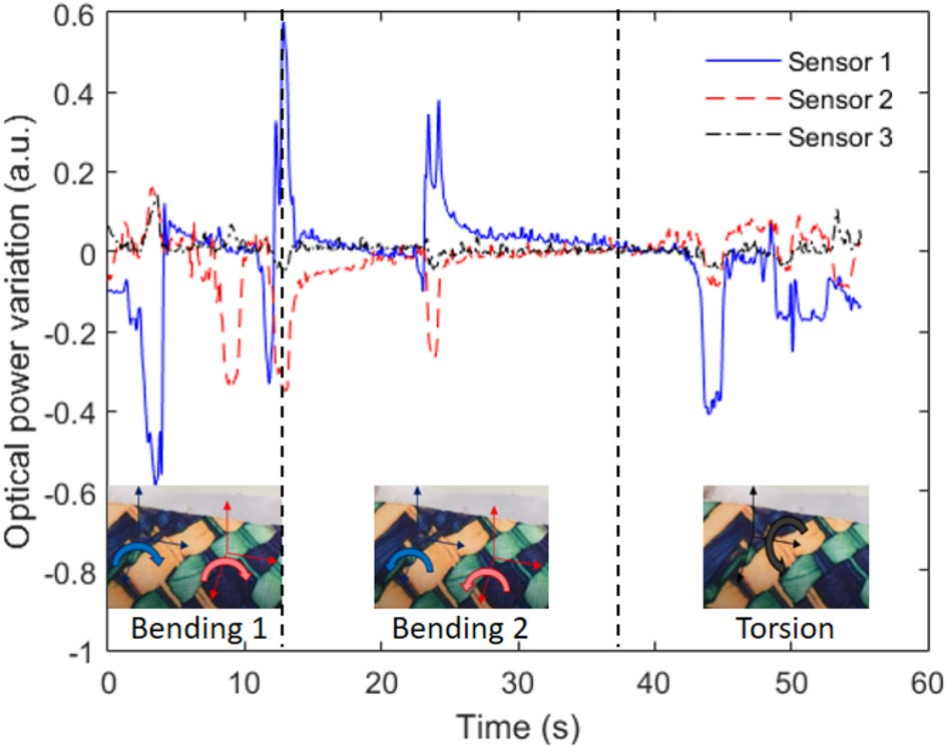
Sensors responses with angular displacement on different planes.

The smart textile sensors responses under displacements applied at different planes are presented in Fig. 5. Bending at different planes and torsions were applied with controlled angles. For the bending, the angles were 0° to 90° and 0° to − 90°, whereas, in the torsion assessment, angular displacements ranging from 0° to 180° were applied, as depicted in Fig. 5 inset. Comparing the responses of each sensor, which are obtained at the angular displacements in different planes, it is possible to observe differences in the sensors behavior, which can be used for the classification of each movement in multiple planes. It is also noteworthy that Sensors 1 and 2 presented the highest bending sensitivity, related to the region where the bending was applied (see Fig. 5 inset), resulting in a higher stress in Sensors 1 and 2. In the torsion case, the highest optical power variation was also obtained in Sensor 1, whereas, once again, the Sensor 3 presented the lowest signal variation. However, the differences in the sensors sensitivities obtained in each case can be used for the estimation of the multiplane displacements applied on the textile, using techniques such as transfer matrix for 3D plane displacements assessment^34^, where a system of equations obtained in the sensors characterizations are used for the angle assessment of each plane. It enables the remote human movement analysis using wearable sensors that do not inhibit the natural pattern of the user’s movements.

As shown in Fig. 3, the sensors are also sensitive to temperature variations. Thus, temperature variations interfere on the bending and force assessment. Although the results presented in Figs. 4 and 5 were obtained in constant temperature conditions, temperature variations can occur in practical applications. In order to mitigate the temperature influence on the sensors’ responses, two approaches are considered. The first approach is based on the difference between sensors responses for temperature and strain-related parameters (as previously validated in temperature-compensated systems^35^), considering their previously characterized sensitivities with respect to temperature, angle and force, in this case. Furthermore, the use of the smart textile in dynamic movement applications leads to an additional possibility of temperature compensation. In practical applications of the proposed textile, the temperature variation rate is lower than the one of the strain-related parameters (such as force and angle). This behavior leads to differences in the frequency components of the temperature and strain, where the lower frequencies are related to the temperature. Therefore, the temperature influence on the strain response is mitigated by filtering the low frequencies components on the sensor responses, as demonstrated in previous works^36^.

It is also worth noting that the smart textile characterization with respect to temperature and force show a high repeatability of the sensors with low standard deviation (0.004 a.u.) in the temperature assessment after 3 sequential tests (see the error bars in Fig. 3a). Moreover, the error bars are not visible in Fig. 4b due to its low standard deviation (0.005 a.u.). As another indicator of the sensors’ consistency, the bending tests, whose results are shown in Fig. 5, show the sensor reversibility, since the sensors responses returned to their initial condition without significant residual strains after the bending is performed. The maximum reversibility error (obtained by the comparison between the sensors responses before and after the bending) is 0.03 a.u., considering all three sensors.

**Figure 6:**
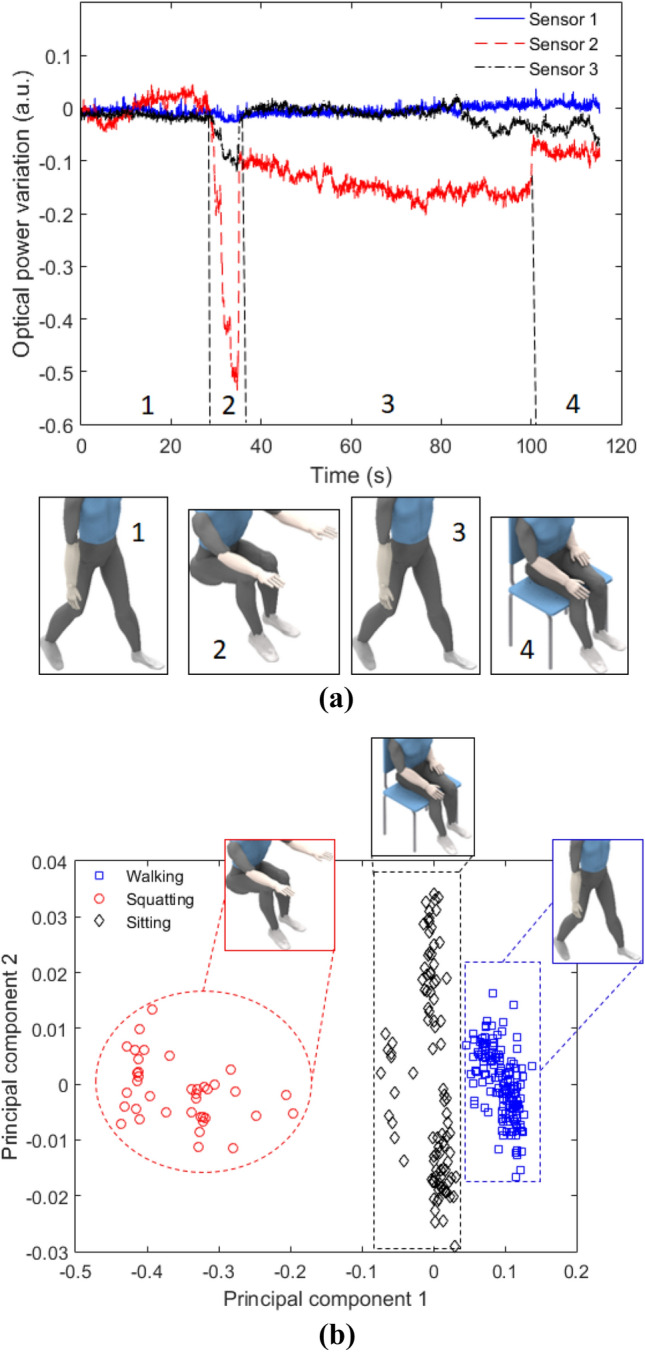
LPS-POF embedded smart textile for user’s activity monitoring. (**a**) Time-domain analysis. (**b**) Scatter plot for the activities monitoring.

In the last evaluation of the smart textile performance, the capability of detecting user’s activities is assessed by means of positioning the textile in a healthy volunteer. The textile is positioned on the user’s lower back. Its positioning in the lower back enables to acquire the signals from the activities characterized by strong correlation with user’s trunk displacement, as lower limb movements generally result in variations on the trunk, the smart textile can be used on the detection of gait-related activities as well. Thus, the user was asked to perform three common movements in daily routing: walking in self-selected speed, squatting and seat on a chair. The sensors responses for each case are presented in Fig. 6a, where the sensors are analyzed in the time domain. The time domain response shows significant differences in the sensors responses, especially in the squatting activity, where Sensor 2 shows the highest signal attenuation, which is related to its positioning at the center of user’s lower back, leading to higher displacements in the fiber when compared with Sensors 1 and 3. Another offset in the sensors’ responses were found when the user sits on a chair, where the back support of the chair resulted in an attenuation on the transmitted optical power. The walking activity is the one that induced the lowest optical power variation in the sensors. In order to enhance the activity detection performance, the PCA was applied in the sensors responses. The PCA represents the data in a new coordinate system by using linear transformations and is a widely used technique for dimensionality reduction and as preprocessing for clustering techniques^37^. In this case, the PCA was applied in the data to show the possibility of activity detection and identification using the proposed smart textile when used in conjunction with a clustering technique such as k-means^38^. Figure 6b shows the scatter plot of first two principal components for the sensors responses obtained on each activity, these two principal components represent 98% of all variability, mainly related to time-domain responses of Sensors 2 and 3, which indicate the possibility of dimensionality reduction by using just the first two principal components (instead of all components). Furthermore, the PCA results in three separate groups, which are identified as three clusters, representing each performed activity. It is important to mention that a higher number of observations was obtained in the walking and sitting activities due to their longer duration when compared with squatting activity, as shown Fig. 6a. Thus, the PCA in conjunction with k-means (or other clustering technique) result in a feasible option for the activities detection using the proposed smart textile. It is also worth noting that the proposed textile has scalability, where a higher number of sensors can be used, which can lead to the detection of a higher number of activities, depending on the sensor system positioning on the user.

The experimental results obtained with the proposed smart textile shows the feasibility of this approach in a novel, multiparameter and transparent sensor system that can be used in multiple applications. It is possible to envisage that the next generation of the textile fabrics will be embedded with sensor system for the assessment of the user’s health condition and activities, where the proposed technology (i.e. LPS-POF embedded in conjunction with multiplexing techniques based on LEDs modulation) can be a key technology for transparent multipurpose sensor systems. The proposed smart textile can be also integrated with IoT modules for remote health monitoring. In addition, due to the scalability of the proposed technique, it is also possible to envisage the development of a complete clothing embedded with the proposed sensors for the assessment of multiple parameters, such as heart and breath rates, kinematic parameters of the human, interaction forces, body temperature, just to name a few. All these sensors transmitting to a home gateway and IoT modules for the remote health monitoring, which can also be integrated with novel classifiers and neural network in order to extract all the physical and physiological information of the user.

## Discussions

In this paper, a novel smart textile based on multiplexed intensity variation-based sensors was proposed. The multiplexing technique is based on an OOK modulation in the LEDs, which results in a quasi-distributed sensor system with low cross-sensitivity. In contrast with other popular quasi-distributed optical fiber sensors, such as FBGs, the proposed approach does not need neither specialized equipment for the sensor fabrication nor high cost (and generally bulk) interrogators for the signal acquisition. Therefore, the proposed technique is fully portable and low cost, which enable a plethora of applications in remote health monitoring by either transparent wearable sensors or smart homes. In addition, the LPS-POF used in the smart textile has remarkable flexibility (Young’s modulus of 15 MPa) in conjunction with high strain limits (about 17%), which can be embedded in clothing without restricting the user’s movement and results in sensors with high sensitivity and can be used in a large range of dynamic movements, as verified in the DMA experiments.

The LPS-POF and flexible LEDs were embedded in between two layers of neoprene textile fabric for the multiplexed sensor system. The temperature and interaction forces characterization showed the high sensitivity and linearity of the sensor system, which can also measure the temperature profile and force map with high repeatability and resolution. Then, the movement analysis using the proposed textile was performed by means of multiplane angle assessment using the textile sensors for bending at different planes as well as torsion, where the sensors presented different sensitivities with the possibility of simultaneous assessment of angular displacement at different planes. Finally, the smart textile was positioned on the user’s lower back and was able of identifying basic activities in daily life such as walking, siting and squatting by the analysis of the sensors responses using PCA in conjunction with clustering techniques. Therefore, the proposed device is a feasible option for novel transparent wearable sensors for remote health monitoring, which can be scalable for a fully instrumented clothing with smart sensors. Future works include the development and validation of the smart clothing for simultaneous and remote evaluation of multiple parameters.

## Methods

### LPS-POF fabrication

The LPS-POF fabrication is performed in three steps: (1) addition of a liquid mixture of monomers and additive in a dosing system, where the main monomer is the Bisphenol-A acrylate due to its combination of high elasticity and optical transparency. (2) The liquid mixture passes through a spinneret in order to obtain the cylindrical shape. (3) The UV-curing is performed for the polymerization followed by an axial deformation stage to obtain the desired diameter for the optical fiber.

### DSC tests

In order to characterize the fiber thermal transitions, DSC is performed, in which the LPS-POF enthalpy variation is compared with a thermally inert reference^39^. The DSC equipment employed is the DSC Q200 (TA Instruments, USA), where the heat flow variation in the sample is analyzed with respect to the temperature. In this test, the offset in the heat flow variation curve is related to the polymer glass transition temperature, whereas an endothermic peak corresponds to its melting temperature.

### Tensile and DMA tests

The tensile tests were performed using a universal testing machine (Biopdi, Brazil) in which the fiber was positioned in the machine’s clamps for the axial strain tests, where the displacement (strain) and force (stress) were continuously monitored. The test occurs until the fiber breakage, where it is possible to infer the strain limits of the LPS-POF. In addition, the Young’s modulus of the fiber is calculated as the ratio between the stress and strain in the elastic region (linear region) of the stress–strain curve. All the tensile tests were made with constant strain rate of 1 mm/min. Then, the DMA tests are performed by means of applying an oscillatory load in the sample with controlled displacement and frequency at constant temperature condition. The DMA 8000 (Perkin Helmer, USA) was used in the experiments in the axial strain configuration with a maximum displacement of 1 mm, where each measurement was performed 3 times in an isothermal period of 10 min. The temperature in the tests was 25 °C.

### Temperature characterization setup

In the temperature characterization tests, Sensors 1, 2 and 3 were positioned in a thermoelectric Peltier plate TEC-12706 (Heibei IT, China) with closed loop temperature control TED 200C (Thorlabs, USA). The temperature range was 20–40 °C in 5 °C steps, where the isothermal period was 5 min in order to ensure a constant temperature at each sensor. For the temperature profile tests, a temperature-controlled hot air blower 858D (QWERTOUY, USA) was employed and positioned at different regions of the smart textile.

### Force characterization setup

For the force characterization, calibrated weights with a known mass were positioned on the top of each sensor for about 10 s. All the sensors were tested in a range of 0–150 N. However, Sensors 1 and 3 have different forces due to the dimensions of the weights, which can apply a force on Sensor 2 due to the proximity of such sensors. In addition, the force map characterization was performed by position a weight with larger dimensions in the center of the smart textile, leading to a force distribution in the sensors.

### Angular displacement characterization setup

The angular displacement tests were performed by manually bending the textile with the aid of a goniometer at the different planes, where the goniometer was previously aligned with the bending plane and the angular displacements in the range of 0° to 90° for bending and 0° to 180° for torsion were performed.

### Human activity monitoring protocol

In the smart textile validation test, a healthy volunteer (male, 29 years) positioned the smart textile in his lower back with the aid of elastic bands. The test comprises of walking in a 5-m room at constant velocity and, at the end of the 5 m walk, the user performs a squat. Then, there is 180° turn and the user returns to the end of the room, where a chair is positioned. The user sits on the chair and the test ends at about 30 s later. We have the informed consent of all participants and the tests were made in accordance with the guidelines of the national health council with the protocols approved by Research Ethics Committee through the National Commission in Research Ethics—CONEP—(Certificate of Presentation for Ethical Appreciation—CAAE: 64797816.7.0000.5542). The responses of all three sensors were acquired for each activity, i.e. walking, squatting and sitting. The signal processing was performed using PCA, since it is a widely used statistical tool for multivariable data analysis (details on the PCA algorithm can be found in Jollife and Cadima^37^), where the data distributed in the principal components can be classified using techniques such as the k-means clustering.
